# Trend in mandatory immunisation coverage: linear and joinpoint regression approach, Serbia, 2000 to 2017

**DOI:** 10.2807/1560-7917.ES.2021.26.26.2000417

**Published:** 2021-07-01

**Authors:** Marko Veljkovic, Goranka Loncarevic, Milena Kanazir, Darija Kisic-Tepavcevic, Tatjana Gazibara

**Affiliations:** 1 Institute of Public Health of Serbia “Dr Milan Jovanovic Batut”, Belgrade, Serbia; 2 Institute of Epidemiology, Faculty of Medicine, University of Belgrade, Belgrade, Serbia

**Keywords:** vaccination, coverage, schedule, trend

## Abstract

**Background:**

Analyses of temporal trends in immunisation coverage may help to identify problems in immunisation activities at specific points in time. These data are essential for further planning, meeting recommended indicators, monitoring, management and advocacy.

**Aim:**

This study examined the trends of mandatory vaccination coverage in the period 2000–2017 in Serbia.

**Methods:**

Data on completed immunisations were retrieved from annual national reports of the Institute of Public Health of Serbia during the period 2000–2017. To assess the trends of immunisation coverage, both linear and joinpoint regression analyses were performed. A probability p < 0.05 was considered significant.

**Results:**

Over the period 2000–2017 linear regression analysis showed a significant decline in coverage with the primary vaccination against poliomyelitis, diphtheria, tetanus, pertussis and measles, mumps, rubella (MMR) (p ≤ 0.01). In the same period, coverage of all subsequent revaccinations significantly decreased, namely, first revaccination for pertussis (p < 0.01); first, second and third revaccination against diphtheria, tetanus and poliomyelitis (p < 0.01); and second dose against MMR before enrolment in elementary school (p < 0.05). Although linear regression analysis did not show change in vaccination coverage trend against tuberculosis (Bacillus Calmette–Guérin; BCG), hepatitis B (HepB3) in infants and diseases caused by *Haemophilus influenzae* type b (Hib3), the joinpoint regression analysis showed that the coverage declined for BCG after 2006, HepB3 after 2010 and Hib3 after 2008.

**Conclusion:**

To achieve and keep optimum immunisation coverage, it is necessary to address barriers to immunisation, such as the availability of all vaccines and vaccine-hesitancy among parents and healthcare workers in Serbia.

## Introduction

Immunisation is one of the most effective tools in primary prevention of communicable diseases. Together with safe drinking water, immunisation plays a key role in efforts to reduce mortality from communicable diseases [1]. The World Health Organization (WHO) estimates suggest that systematic immunisation helps to prevent 2–3 million deaths each year [2].

According to the Law on Population Protection from Communicable Diseases [3], immunisation against 11 infectious diseases is currently mandatory in Serbia. These diseases include tuberculosis, diphtheria, tetanus, pertussis, poliomyelitis, diseases caused by *Haemophilus influenzae* type b (Hib), hepatitis B (hepB), measles, mumps, rubella (MMR) and, as of April 2018, pneumococcal disease. Mandatory immunisation is carried out continuously and in agreement with the vaccination schedule, unless temporary or permanent contraindications are identified [3,4]. All mandatory vaccines are provided free of charge.

While systematic immunisation of population in Serbia has been a long-standing prevention strategy against communicable diseases [5], the main barriers to optimum immunisation since 2000 have included limited availability of vaccines and occasional interruption of vaccine supply [6] as well as vaccine-hesitancy [7]. Analyses of temporal trends in immunisation coverage may help public health authorities identify problems in immunisation activities at specific points in time. These data are essential for further planning, meeting recommended indicators, monitoring, management and advocacy [8]. Finally, because of resurgence of some vaccine-preventable diseases (VPDs) in Serbia, such as measles [9,10], analysis of temporal trends of immunisation coverage could help to detect those birth cohorts that require supplementary immunisation to improve vaccination programmes and ensure their optimum performance.

The purpose of this study was to examine the trend in mandatory immunisation coverage in the period 2000–2017 in Serbia.

## Methods

### Data collection and coverage

Data about immunisation coverage from 2000 to 2017 were based on the annual national reports on completed immunisations. The reports were available from the centralised database at the Institute of Public Health of Serbia “Dr Milan Jovanovic Batut”.

The national-level reports were based on records of 23 district (local) institutes of public health in Serbia (Supplemental material). The local institutes of public health are in charge of collecting and compiling information about immunisation in corresponding districts. The districts are composed of municipalities. Each municipality has one community health centre, which provides primary healthcare to the municipality residents including immunisations [4]. Community health centres also supply district institutes of public health with relevant health data. Children have individual health records in their municipal community health centre (paediatric department). Information about health status and the vaccines received by each child is entered in their health records.

The coverage rate for each calendar year was calculated as the proportion of the immunised persons in a birth cohort targeted for immunisation [11]. The numerator represented the number of children who received the specific vaccine during the observed calendar year. These data were retrieved from paper-based and electronic health records. The denominator represented the size of the population targeted for immunisation who had a health record in the municipal community health centre i.e. the number of children in a birth cohort intended to receive vaccines corresponding to the immunisation schedule for that particular calendar year.

### Ethical statement

Because this study used secondary data, collected from the regular and mandatory reports, and did not include human or animal participants, it was exempt from ethics review by the Ethics Committee of the Institute of Public Health of Serbia.

### Immunisation schedule

In the period 2000–2005, mandatory immunisation had been conducted against eight communicable diseases: tuberculosis, diphtheria, tetanus, pertussis, poliomyelitis, MMR. Vaccines against hepB and diseases caused by Hib were introduced in the national mandatory immunisation schedule in 2005 and 2006, respectively [4]. The most recent modification of the national mandatory immunisation schedule was made in 2018, when the pneumococcal conjugate vaccines (PCV) were included [3], with three doses of PCV (PCV10, PCV13) for primary vaccination at age 2–6 months and one dose at age 2 years for revaccination. Given the time period chosen for this study, pneumoccocal conjugate vaccination will not be included in the analysis. The immunisation schedule during the period 2000–2017 is presented in the Table.

**Table:** Mandatory immunisation programme, Serbia, 2000–2017 (n = 10 diseases)

Disease		Vaccine	Number of doses	Age/period of life	Comment
Tuberculosis		BCG vaccine	1	At birth	None
Hepatitis B		Hep B vaccine	3	At birth, 1, 6** **months	Introduced in 2005
				12** **years^a^	Introduced in 2006
Diphtheria, tetanus, pertussis, poliomyelitis, diseases caused by Hib	Primary vaccination	DTP vaccine	3	2–6 months	None
		tOPV			None
		Hib vaccine			Hib was introduced in 2006
		DTaP-IPV-Hib			DTaP-IPV-Hib was introduced in 2015 instead of DTP, tOPV, Hib
	First revaccination	DTP vaccine	1	18–24 months	None
		tOPV			None
		DTaP-IPV-Hib			DTaP-IPV-Hib was introduced in 2015 instead of DTP, tOPV, Hib
	Second revaccination	DT vaccine	1	Before enrolment in elementary school	None
		tOPV/bOPV			bOVP was introduced instead of tOPV in 2016
	Third revaccination	dT vaccine	1	14 years	None
		tOPV/bOPV			bOVP was introduced instead of tOPV in 2016
MMR	Primary vaccination (1^st^ dose)	MMR vaccine	1	12–15 months	2^nd^ dose of MMR vaccine has been administered at 12 years of age since introduction in 1994; In 2006 2^nd^ dose of MMR vaccine was also introduced before enrolment in elementary school; In the period 2006–2011, 2^nd^ dose was administered both to children aged 12 years (born between 1994 and 1999) and before enrolment in elementary school (children born in 2000 and later).
	Revaccination (2^nd^ dose)	MMR vaccine	1	Before enrolment in elementary school (commonly, ages 6 or 7 years)	
				12 years	

BCG: Bacillus Calmette–Guérin vaccine; bOPV: bivalent oral poliovaccine; DT: diphtheria and tetanus toxoids booster for children; dT: diphtheria and tetanus toxoids booster for adults; DTaP-IPV-Hib: diphtheria and tetanus toxoids, acellular pertussis, inactivated poliovirus and conjugated vaccine against diseases caused by Hib; DTP: diphtheria and tetanus toxoids, whole-cell pertussis vaccine; Hep B: monovalent vaccine against hepatitis B; Hib: *Haemophilus influenzae* type b; Hib vaccine: monovalent conjugated vaccine against diseases caused by Hib; MMR: measles, mumps and rubella; tOPV: trivalent oral poliovaccine.

^a^ Catch-up vaccination with three doses for children who did not receive primary vaccination in infancy.

### Data analysis

Statistical analysis was performed in the Statistical Package for Social Sciences, version 23.0 for Windows (SPSS inc. Chicago, Illinois, United States). The linear regression equation was used to estimate the trend for each vaccine in the national programme. The F test was performed to assess the probability level of the linear regression coefficient. In this equation, the dependent variable is the achieved immunisation coverage for each given year, while the independent variable is time. The probability level (p) of < 0.05 was considered significant.

We further analysed the trend of mandatory vaccination coverage using the joinpoint regression analysis. This analysis provides a more detailed insight into dynamics of the trend of coverage. Specifically, we were able to identify the time points (i.e. joinpoints) over the observed period in which a significant change in trend emerged. The joinpoint regression analysis was performed using an open-source software version 4.7.0.0 [12]. The independent variable was time (i.e. the interval between the year of the first and the last reported vaccination coverage). The dependent variables were percentages per year (coverage) for each vaccine in the immunisation programme (Supplementary material Table S1). We assumed that the error variance was constant and we did not log-transform our data. The maximum number of joinpoints was predefined based on the number of data points. Given that we analysed the immunisation coverage over a period of 18 years, the maximum number of joinpoints was limited to three [12]. The p level of 0.05 was taken as the upper limit of statistical significance.

## Results

**Figure 1:**
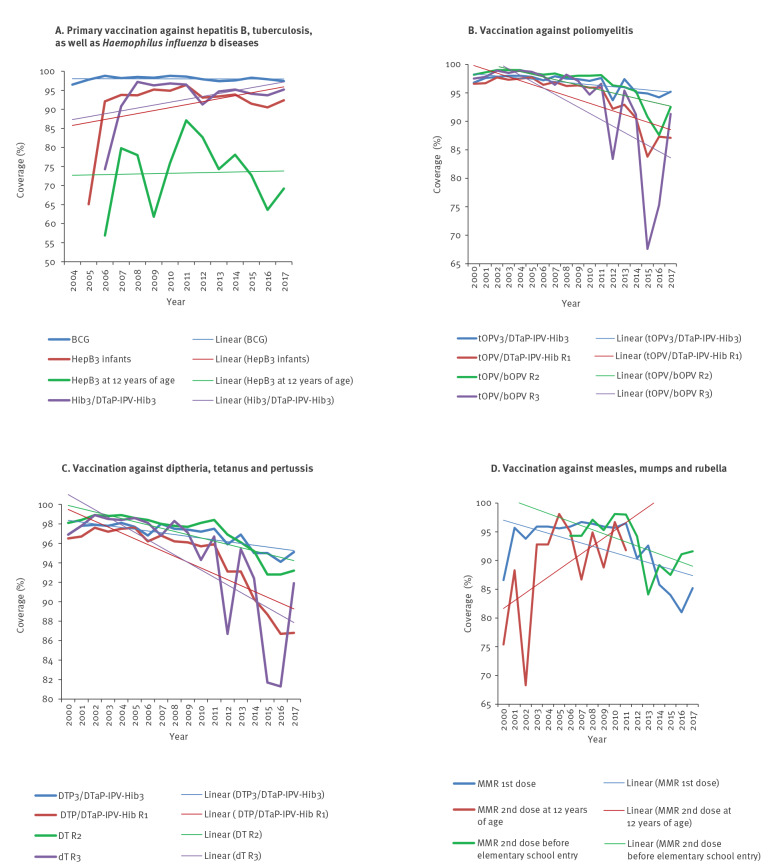
Mandatory immunisation coverage for 10 vaccine-preventable diseases according to disease and vaccination doses, Serbia, 2000–2017

Figure 1 shows vaccination coverage trend for 10 VPDs in the mandatory vaccination programme from 2000 to 2017. The lowest coverage for the vaccine against tuberculosis (Bacillus Calmette–Guérin; BCG) of 96.5% was observed in 2004 (Figure 1A). The lowest coverages with three doses of monovalent HepB (HepB3) and Hib (Hib3) vaccines were observed in the years when these vaccines were introduced in the calendar (2005 and 2006, respectively). The lowest coverage for primary vaccination against poliomyelitis was registered in 2012 (93.7%; Figure 1B), as monitored through the coverage by three doses of trivalent oral poliovirus (tOPV3) and by three doses of diphtheria and tetanus toxoids (DT) acellular pertussis, inactivated poliovirus and conjugated vaccine against diseases caused by Hib (DTaP-IPV-Hib3). The lowest coverage for primary vaccination against diphtheria, tetanus and pertussis was observed in 2016 (94.1%; Figure 1C), as assessed by the combined coverage of the DT whole-cell pertussis vaccine (DTP3) and the DTaP-IPV-Hib3. The year 2016 was also the year with the lowest coverage for the first dose of the MMR vaccine (81%; Figure 1D). The per cent coverage for all 10 VPDs per year is presented in Supplementary material Table S1.

BCG: Bacillus Calmette–Guérin vaccine; bOPV: bivalent oral poliovaccine; DT: diphtheria and tetanus toxoids booster for children; dT: diphtheria and tetanus toxoids booster for adults; DTaP-IPV-Hib: diphtheria and tetanus toxoids, acellular pertussis, inactivated poliovirus and conjugated vaccine against diseases caused by *Haemophilus influenzae* type b; DTP: diphtheria and tetanus toxoids whole-cell pertussis vaccine; Hep B: monovalent vaccine against hepatitis B; MMR: measles, mumps and rubella; tOPV: trivalent oral poliovaccine, R1: first revaccination; R2: second revaccination; R3: third revaccination.

A. Primary vaccination against tuberculosis (BCG), hepatitis B with three doses of HepB (HepB3) among infants and at age 12 years and against diseases caused by Hib with three doses of Hib vaccine (Hib3). The monitoring of BCG vaccination coverage from 2000 to 2003 was organised by the National Institute of Lung Diseases so data on BCG vaccination coverage in the National Institute of Public Health of Serbia are available from 2004. HepB3 vaccination for infants was introduced in 2005. HepB3 at 12 years of age as well as Hib vaccination were introduced in 2006.

B. Primary vaccination against poliomyelitis (three vaccine doses) with tOPV3/DTaP-IPV-Hib3, as well as first (tOPV/DTaP-IPV-Hib R1), second (tOPV/bOPV R2) and third (tOPV/bOPV R3) revaccination.

C. Primary vaccination against diphtheria, tetanus and pertussis (three doses) with DTP3/DTaP-IPV-Hib3 and first revaccination (DTP/DTaP-IPV-Hib R1), as well as second (DT R2) and third (dT R3) revaccination for diphtheria and tetanus.

D. Vaccination against MMR. The second dose of MMR vaccine has been administered at 12 years of age since the introduction of the vaccine in 1994. In 2006 the second dose of MMR was also provided before enrolment in elementary school. During the period 2006–2011 the second dose of MMR was administered both in children aged 12 years (born between 1994 and 1999) and before enrolment in elementary school (children born between 2000 and 2005). After 2011, the second dose of MMR was administered only before enrolment in elementary school.

Over the period 2000–2017 linear regression analysis showed a significant decline in coverage for the primary vaccination against poliomyelitis (p < 0.01; Figure 1B), diphtheria, tetanus, pertussis (p < 0.01; Figure 1C), and MMR (p = 0.01; Figure 1D). In the same period, coverage of all subsequent revaccinations significantly decreased (namely, first revaccination for pertussis (p < 0.01); first, second and third revaccination against diphtheria, tetanus and poliomyelitis (p < 0.01); and second dose of MMR before enrolment in elementary school (p < 0.05)).

**Figure 2:**
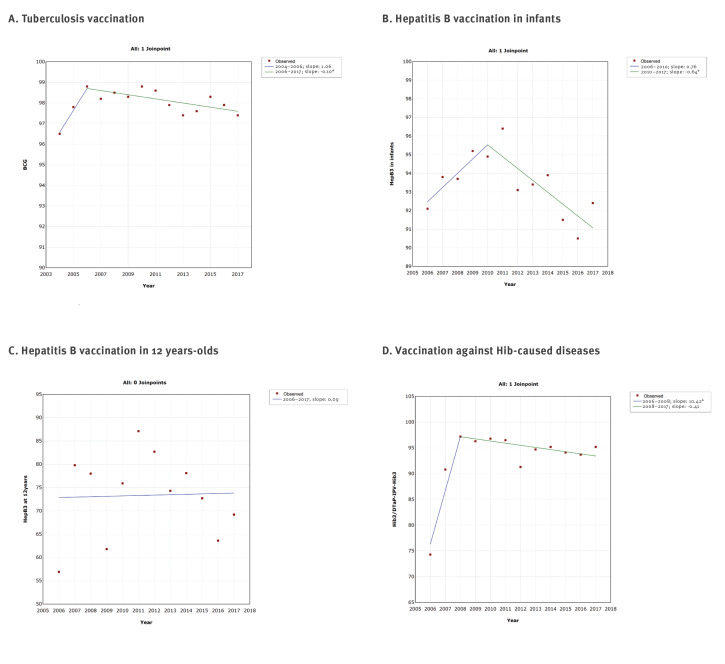
Graphical display of joinpoint regression analysis of vaccination coverage against tuberculosis, hepatitis B and diseases caused by *Haemophilus influenzae* b, according to vaccines, Serbia, 2000–2017

BCG: Bacillus Calmette–Guérin vaccine; DTaP-IPV-Hib: diphtheria and tetanus toxoids, acellular pertussis, inactivated poliovirus and conjugated vaccine against diseases caused by *Haemophilus influenzae* type b; Hep B: monovalent vaccine against hepatitis B.

**Figure 3:**
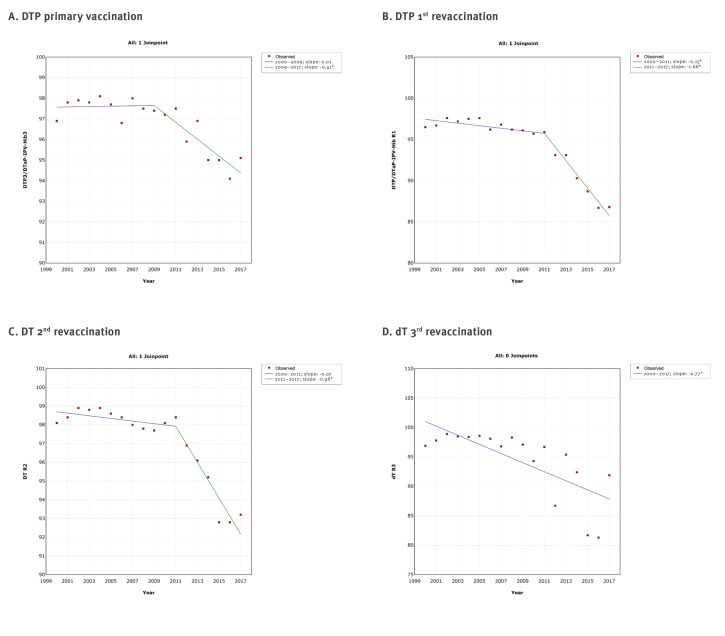
Graphical display of joinpoint regression analysis of vaccination coverage against diphtheria, tetanus and pertussis with time, according to vaccines and doses, Serbia, 2000–2017

DT: diphtheria and tetanus toxoids booster for children; dT: diphtheria and tetanus toxoids booster for adults; DTaP-IPV-Hib: diphtheria and tetanus toxoids, acellular pertussis, inactivated poliovirus and conjugated vaccine against diseases caused by *Haemophilus influenzae* type b; DTP: diphtheria and tetanus toxoids whole-cell pertussis vaccine; R1: first revaccination; R2: second revaccination; R3: third revaccination.

**Figure 4:**
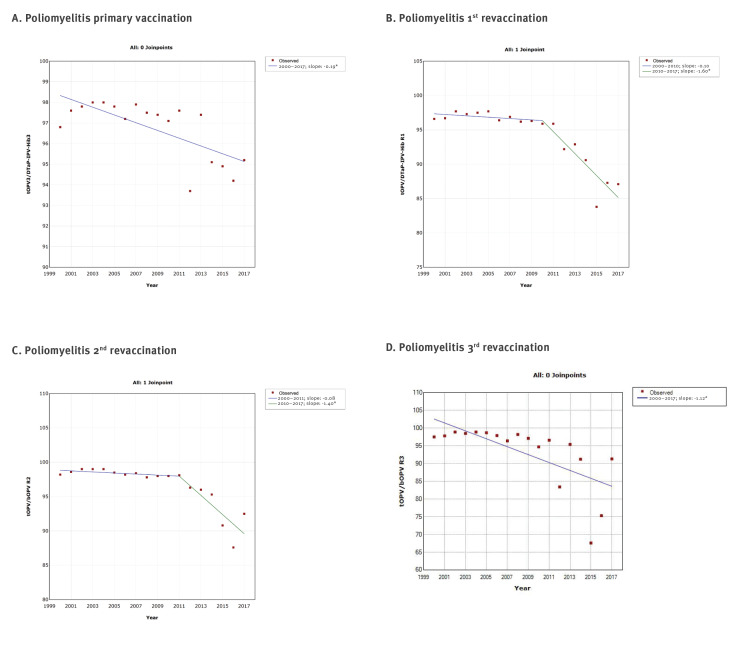
Graphical display of joinpoint regression analysis of vaccination coverage against poliomyelitis with time, according to vaccines and doses, Serbia, 2000–2017

**Figure 5:**
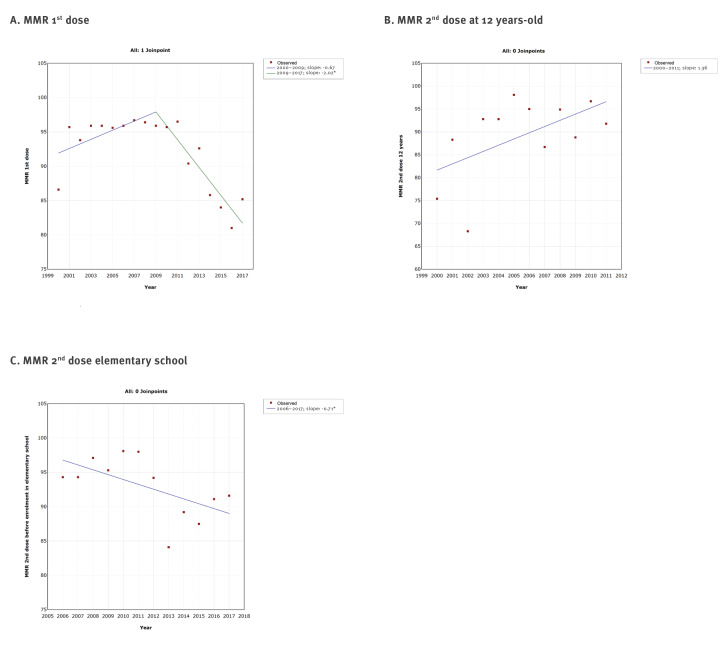
Graphical display of joinpoint regression analysis of vaccination coverage against measles, mumps and rubella with time, according to vaccines and doses, Serbia, 2000–2017

The joinpoint regression analysis showed at a maximum one joinpoint for all vaccines from the immunisation programme (Figures 2,3,4,5). Joinpoints were observed for BCG, Hep B3 for infants, Hib3/DTaP-IPV-Hib3 (Figure 2), DTP3/DTaP-IPV-Hib3, DTP/DTaP-IPV-Hib R1, DT R2 (Figure 3), tOPV/DTaP-IPV-HibR1, tOPV/bOPVR2 (Figure 4) and first dose of MMR vaccine (Figure 5).

* Denotes that the slope for the observed period significantly differed (p < 0.05) from its predecessor.

A. The monitoring of BCG vaccination coverage from 2000 to 2003 was organised by the National Institute of Lung Diseases so data on BCG vaccination coverage in the National Institute of Public Health of Serbia are available from 2004.

B. HepB3 vaccination for infants (i.e. vaccination with three doses of HepB) was introduced in 2005, however during that year there were administrative difficulties to carry out the immunisation so systematic immunisation began the following year.

C. HepB3 at 12 years of age was introduced in 2006.

D. Hib vaccination was introduced in 2006.

* Denotes that the slope for the observed period significantly differed (p < 0.05) from its predecessor.

bOPV: bivalent oral poliovaccine; DTaP-IPV-Hib: diphtheria and tetanus toxoids, acellular pertussis, inactivated poliovirus and conjugated vaccine against diseases caused by *Haemophilus influenzae* type b; R1: first revaccination; R2: second revaccination; R3: third revaccination; tOPV: trivalent oral poliovaccine.

* Denotes that the slope for the observed period significantly differed (p < 0.05) from its predecessor.

MMR: measles, mumps and rubella vaccine.

* Denotes that the slope for the observed period significantly differed (p < 0.05) from its predecessor.

The second dose of MMR has been administered at 12 years of age since the introduction of the vaccine in 1994. In 2006 the second dose of MMR was also provided before enrolment in elementary school. During the period 2006–2011 the second dose of MMR was administered both in children aged 12 years (born between 1994 and 1999) and before enrolment in elementary school (children born between 2000 and 2005). After 2011, the second dose of MMR was administered only before enrolment in elementary school.

This analysis confirmed the downward trend in vaccination coverage against diphtheria, tetanus and pertussis (Figure 1C and Figure 3), poliomyelitis (Figure 1B and Figure 4) and MMR (Figure 1D and Figure 5). However, it is interesting to note that joinpoints (i.e. significant decline) were observed for trends of BCG, HepB3 and Hib3 (Figure 2), for which the linear regression analysis (Figure 1A) did not show overall change from 2000 to 2017. Specifically, compared with period 2004–2006, the coverage of BCG after 2006 significantly decreased (Figure 2A). For Hep B3, compared with 2006–2010, a significant decline was observed after 2010 (Figure 2B). For Hib3, we observed significant decrease after 2008 compared with period 2006–2008 (Figure 2D). As per results of the joinpoint analysis, the primary vaccination coverage with DTP3/DTaP-IPV-Hib3 (Figure 3A) and MMR began decreasing from 2009 onward (Figure 5A).

## Discussion

In this study, linear regression analyses were conducted on vaccine coverage between 2000 and 2017 for 10 VPDs against which immunisation is mandatory in Serbia. A significant decrease in overall coverage for seven of these VPDs including diphtheria, tetanus, pertussis, poliomyelitis, measles, mumps and rubella was found during this period. A joinpoint analysis also showed a decline in vaccination coverage against three additional VPDs (tuberculosis, hepatitis B and diseases caused by Hib) that was not detected by linear regression. The decline for primary vaccination coverage against these VPDs was after 2006 for BCG, after 2010 for HepB3 and after 2008 for Hib3 vaccine.

Our findings are in line with the downward trend in the average immunisation coverage against diphtheria, tetanus, pertussis, poliomyelitis and measles among children aged 2 years in Europe for the period 2009−2017 [13]. In an attempt to remedy this decline, the passing of new laws on mandatory immunisation in some European countries (Italy and France) subsequently resulted in an increase of vaccination coverage [14-16].

In Serbia, diphtheria was eliminated in 1980 and poliomyelitis was eradicated in 2000 [6]. Nevertheless, the observed decline in vaccine coverage in the country may pose a risk for re-emergence of diphtheria [17]. Furthermore, the low coverage of poliomyelitis vaccine may threaten the polio-free status of Serbia and lead to a situation similar to that in Bosnia and Herzegovina and Romania, which have been deemed at risk of a polio outbreak by the European Regional Commission for Certification of Poliomyelitis Eradication due to inadequate performance of the immunisation programmes [18].

Despite efforts to eliminate measles in at least five WHO regions by 2020 [19], the number of measles cases in 2018 globally increased by 167% compared with 2016 [20]. A worldwide increase in estimated measles-related mortality rate has also been registered since 2017 [20]. In Serbia, the most recent measles outbreak was reported in October 2017 [21], ca 1 year after the coverage of MMR vaccine reached its minimum in the country according to the results of the current study. By the end of August 2019, a total of 5,798 measles cases were reported mostly among children under 5 years old and adults above 30 years of age. One third of the affected were hospitalised, of which 15 died. Almost all persons were either not vaccinated, did not complete the recommended 2-dose vaccination schedule or their measles immunisation status was not known [21].

To achieve and maintain the optimum vaccination coverage, three key components need to be met: preparedness of healthcare institutions to successfully carry out immunisation activities, availability of immunisation services and intention of parents to vaccinate their children [22]. Preparedness of healthcare facilities to perform immunisation depends on the continuous vaccine supply, adequate capacity for vaccine storage and skilled staff [22]. The financial sustainability of the national immunisation programme, based on long-term funding (both local and external) as well as the efficient use of the available resources, has a crucial role in supporting efforts to achieve national, regional and global goals in the realm of prevention of communicable diseases [23]. One of the reasons for the observed decline in immunisation coverage in Serbia was the interruption of vaccine supplies and limited availability of vaccines, which resulted in the occasional disruption of immunisation activities at a national level, particularly during 2012–2015. In these circumstances, the primary vaccination was considered a priority compared with revaccination, which can partially explain the decline in revaccination coverage, such as for MMR and OPV. Similarly, disrupted supply of Hep B and Hib vaccines in Serbia in the first years after they had been introduced in the immunisation programme [24,25] could explain the lowest coverage rates observed in those years.

Availability of immunisation services depends on resources allocated for immunisation activities as well as on physical access to healthcare facilities where immunisation is routinely performed [22]. These conditions are almost entirely met by the Serbian healthcare system, because of the organisation of primary healthcare delivery through community health centres located in all municipalities in Serbia. Therefore, almost all residents have relatively easy access to healthcare stations and posts even in remote rural areas. This, combined with public funding of the vaccines from the immunisation programme allow all children universal access to primary healthcare.

Immunisation coverage also depends on parents’ intention to comply with vaccination [22]. One potential reason for the downward trend in immunisation coverage in Serbia, particularly over the last years, is related to parental vaccine hesitancy [7]. According to the WHO, vaccine hesitancy has been acknowledged as a major threat to global health in 2019 [26], because of delay or refusal of vaccination despite the availability of vaccines and immunisation services [27]. Several factors have been associated with vaccine hesitancy, such as confidence (i.e. trust in the effectiveness, safety or delivery of vaccines including healthcare services and healthcare workers), perceived invulnerability (i.e. the perception that VPDs do not represent a health risk) and convenience (i.e. the availability, accessibility, timeliness, affordability and real or perceived quality of immunisation services) [27].

Lack of accurate information, including that on risks and benefits of vaccination, is a major contributor to low confidence in immunisation activities. To increase immunisation coverage, promotion of vaccination needs to be grounded in public confidence in vaccines, health authorities and the healthcare system. In fact, a dialogue between health authorities and the public represents an important element in building trust and raising awareness about resurgence of VPDs. This also includes contemporary communication technologies, such as social media and online platforms [28-30].

Although inadequate communication with healthcare workers may influence parental decision to refuse vaccination [31], recent challenges in promotion of immunisation are related to vaccine hesitancy among healthcare workers [32]. This is a major drawback, because healthcare workers represent the primary source of health information to parents and the lay public. For this reason, continuous education of the healthcare staff about recent advances and research in the field of vaccines is needed. In addition to this, healthcare staff is in need of training focused on communication and delivery of scientific information to parents who are hesitant about vaccination [33]. Identification of healthcare workers who are hesitant about vaccination could allow to define specific concerns and reasons for hesitancy among healthcare personnel. This may help to improve continuous medical education programmes as well as to develop strategies that address vaccine hesitancy.

Vaccine hesitancy may vary over time, between various cultural settings and may be related to different vaccines. Because of this, it is important to take into consideration the local context and specific concerns to overcome vaccine hesitancy [34]. For example, social mobilisation, mass media, non-financial incentives, communication-tool-based training for healthcare workers and reminder/recall-based interventions have been particularly effective in efforts to reduce vaccine hesitancy among parents [35]. Still, comprehensive empirical research focused on vaccine hesitancy determinants both among parents and among healthcare workers in Serbia remains limited.

This study has certain limitations. The national electronic immunisation registry is still being developed. For this reason, numerators and denominators, used to calculate the immunisation coverage, were based on aggregated data from community health centres. The denominators covered only those children in specific birth cohorts who had health records in community health centres. While residents of Serbia have universal healthcare access and mandatory immunisation is provided free of charge, there is a certain percentage of children who receive immunisation in the private healthcare sector. Immunisation records of children who were immunised in private healthcare institutions were not included in this study. The data about primary vaccination coverage included only children who completed vaccination with three doses, while data on incomplete (i.e. children who received only one or two of these doses) or catch-up vaccination were not available. Use of straightforward linear regression is a rather simplistic approach to evaluate trends. For this reason, we applied the joinpoint regression to study more in-depth the dynamics of vaccination coverage over time that cannot be otherwise observed using linear regression.

### Conclusion

This study found that from 2000 to 2017 vaccination coverage in Serbia against diphtheria, tetanus, pertussis, poliomyelitis, measles, mumps and rubella significantly decreased. To achieve and maintain adequate immunisation coverage (i.e. over 95%), it is imperative to have a secure continuous and timely supply of vaccines from the national immunisation programme. Relevance of immunisation defined by the national immunisation programme needs to be addressed repeatedly both through the media and in the healthcare setting. Understanding factors that contribute to vaccine-hesitancy among parents and healthcare workers in Serbia could help to define specific challenges to overcome in the local context. Such information is needed as the baseline framework to tailor specific interventions to overcome vaccine hesitancy.

## Supplementary Data

227000 Supplement
