# Supplementary material for: Trend in mandatory immunisation coverage: linear and joinpoint regression approach, Serbia, 2000 to 2017
Source: Euro Surveill. 2021 Jul 1;26(26):2000417. doi: 10.2807/1560-7917.ES.2021.26.26.2000417 (PMC8326657; doi:10.2807/1560-7917.ES.2021.26.26.2000417)
Supplement: Supplement [file FINAL_Supplemental_material.pdf]

Trend in mandatory immunisation coverage: linear and joinpoint regression approach, Serbia, 2000 to 2017

Supplementary material

This supplementary material is hosted by Eurosurveillance as supporting information alongside the article "Trend in mandatory immunisation coverage: linear and joinpoint regression approach, Serbia, 2000 to 2017", on behalf of the authors, who remain responsible for the accuracy and appropriateness of the content. The same standards for ethics, copyright, attributions and permissions as for the article apply. Supplements are not edited by Eurosurveillance and the journal is not responsible for the maintenance of any links or email addresses provided therein.

District (local) Institutes of Public Health include:

1) Institute of Public Health in Subotica
2) Institute of Public Health in Zrenjanin
3) Institute of Public Health in Kikinda
4) Institute of Public Health in Pancevo
5) Institute of Public Health in Sombor
6) Institute of Public Health of Vojvodina
7) Institute of Public Health in Sremska Mitrovica
8) Institute of Public Health in Sabac
9) Institute of Public Health in Valjevo
10) Institute of Public Health in Pozarevac
11) Institute of Public Health in Kragujevac
12) Institute of Public Health in Cuprija
13) Institute of Public Health in Zajecar
14) Institute of Public Health in Uzice
15) Institute of Public Health in Cacak
16) Institute of Public Health in Kraljevo

17) Institute of Public Health in Krusevac
18) Institute of Public Health in Nis
19) Institute of Public Health in Pirot
20) Institute of Public Health in Leskovac
21) Institute of Public Health in Vranje
22) Institute of Public Health in Kosovska Mitrovica
23) Institute of Public Health in Belgrade

Supplementary Table S1 Mandatory immunization coverage in the Republic of Serbia during the period 2000-2017 (calculated as the proportion of vaccinated children in the specific age group (i.e. birth cohort) intended to receive vaccines according to the National immunization schedule in that particular year)

Year	Coverage (%)													
	BCG	tOPV3 / DTaP-IPV-Hib3	tOPV/ DTaP-IPV-Hib R1	tOPV/ bOPV R2	tOPV/ bOPV R3	DTP3/ DTaP-IPV-Hib3	DTP/ DTaP-IPV-Hib R1	DT R2	dT R3	MMR 1st dose	MMR 2nd dose 12y	MMR 2nd dose before enrolment in elementary school	Hep B3 infants	Hep B3 12y	Hib3/ DTaP-IPV-Hib3
2000	ND	96.8	96.6	98.2	97.5	96.9	96.5	98.1	96.9	86.6	75.4	NA	NA	NA	NA
2001	ND	97.6	96.7	98.6	97.8	97.8	96.7	98.4	97.8	95.7	88.3	NA	NA	NA	NA
2002	ND	97.8	97.7	99.0	98.9	97.9	97.6	98.9	98.9	93.8	68.3	NA	NA	NA	NA
2003	ND	98.0	97.3	99.0	98.5	97.8	97.2	98.8	98.5	95.9	92.8	NA	NA	NA	NA
2004	96.5	98.0	97.5	99.0	98.9	98.1	97.5	98.9	98.4	95.9	92.8	NA	NA	NA	NA
2005	97.8	97.8	97.7	98,5	98.7	97.7	97.6	98.6	98.6	95.6	98.1	NA	65.1	NA	NA
2006	98.8	97.2	96.4	98.2	97.9	96.8	96.2	98.4	98.1	95.9	95.0	94.3	92.1	56.9	74.3
2007	98.2	97.9	96.9	98.4	96.4	98.0	96.8	98.0	96.8	96.7	86.7	94.3	93.8	79.8	90.8
2008	98.5	97.5	96.2	97.8	98.2	97.5	96.2	97.8	98.3	96.4	94.9	97.1	93.7	78.0	97.2
2009	98.3	97.4	96.3	98.0	97.1	97.4	96.1	97.7	97.1	95.9	88.8	95.3	95.2	61.8	96.3
2010	98.8	97.1	95.9	98.0	94.7	97.2	95.7	98.1	94.3	95.7	96.7	98.1	94.9	75.9	96.8
2011	98.6	97.6	95.9	98.1	96.6	97.5	95.9	98.4	96.7	96.5	91.8	98.0	96.4	87.1	96.5
2012	97.9	93.7	92.2	96.3	83.4	95.9	93.1	96.9	86.7	90.4	NA	94.2	93.1	82.7	91.3
2013	97.4	97.4	92.9	96.0	95.4	96.9	93.1	96.1	95.4	92.6	NA	84.1	93.4	74.3	94.7
2014	97.6	95.1	90.6	95.3	91.2	95.0	90.3	95.2	92.4	85.8	NA	89.2	93.9	78.1	95.2
2015	98.3	94.9	83.8	90.8	67.6	95.0	88.7	92.8	81.7	84.0	NA	87.5	91.5	72.7	94.1
2016	97.9	94.2	87.3	87.6	75.3	94.1	86.7	92.8	81.3	81.0	NA	91.1	90.5	63.6	93.7
2017	97.4	95.2	87.1	92.5	91.3	95.1	86.8	93.2	91.9	85.2	NA	91.6	92.4	69.2	95.2

BCG – *Bacillus Calmette–Guérin* vaccine; bOPV – bivalent oral poliovaccine; DTP – diphtheria and tetanus toxoids, whole-cell pertussis; DTaP-IPV-Hib – diphtheria and tetanus toxoids, acellular pertussis, inactivated poliovirus and conjugated vaccine against diseases caused by *Hib*; DT – diphtheria and tetanus toxoids booster for children, dT – diphtheria and tetanus toxoids booster for adults; Hep B – monovalent vaccine against hepatitis B; Hib – monovalent conjugated vaccine against diseases caused by *Haemophilus influenzae* type b; MMR – vaccine against measles, mumps and rubella; tOPV – trivalent oral poliovaccine.

ND – no data; NA- not applicable;

Number 3 denotes the number of doses used in primary vaccination; R1 – first revaccination; R2 – second revaccination; R3 – third revaccination.
